# Property Criteria for Automotive Al-Mg-Si Sheet Alloys

**DOI:** 10.3390/ma7075047

**Published:** 2014-07-04

**Authors:** Ramona Prillhofer, Gunther Rank, Josef Berneder, Helmut Antrekowitsch, Peter J. Uggowitzer, Stefan Pogatscher

**Affiliations:** 1 AMAG Rolling GmbH, Lamprechtshausnerstraße 61, 5282 Ranshofen, Austria; E-Mails: gunther.rank@amag.at (G.R.); josef.berneder@amag.at (J.B.); 2 Institute of Nonferrous Metallurgy, Montanuniversität Leoben, Franz-Josef-Straße 18, 8700 Leoben, Austria; E-Mail: helmut.antrekowitsch@unileoben.ac.at; 3 Laboratory of Metal Physics and Technology, Department of Materials, ETH Zurich, Vladimir-Prelog-Weg 4, 8093 Zürich, Switzerland; E-Mail: peter.uggowitzer@mat.ethz.ch

**Keywords:** Al-Mg-Si alloys, automotive sheets, formability, roping, corrosion

## Abstract

In this study, property criteria for automotive Al-Mg-Si sheet alloys are outlined and investigated in the context of commercial alloys AA6016, AA6005A, AA6063 and AA6013. The parameters crucial to predicting forming behavior were determined by tensile tests, bending tests, cross-die tests, hole-expansion tests and forming limit curve analysis in the pre-aged temper after various storage periods following sheet production. Roping tests were performed to evaluate surface quality, for the deployment of these alloys as an outer panel material. Strength in service was also tested after a simulated paint bake cycle of 20 min at 185 °C, and the corrosion behavior was analyzed. The study showed that forming behavior is strongly dependent on the type of alloy and that it is influenced by the storage period after sheet production. Alloy AA6016 achieves the highest surface quality, and pre-ageing of alloy AA6013 facilitates superior strength in service. Corrosion behavior is good in AA6005A, AA6063 and AA6016, and only AA6013 shows a strong susceptibility to intergranular corrosion. The results are discussed below with respect to the chemical composition, microstructure and texture of the Al-Mg-Si alloys studied, and decision-making criteria for appropriate automotive sheet alloys for specific applications are presented.

## 1. Introduction

Aluminum alloys have attracted considerable interest from the automotive industry in the last few years as manufacturers seek to design lightweight vehicles with improved fuel efficiency and reduced vehicle emissions. Heat treatable Al-Mg-Si alloys (6xxx series) are in particular increasingly used in automotive applications.

**Figure 1:**
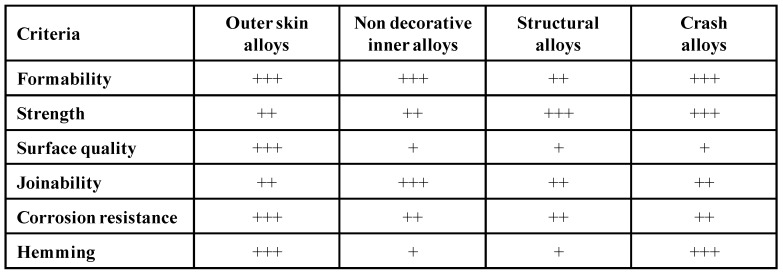
Property criteria for Al-Mg-Si sheet alloys for body-in-white applications [2,3].

Materials for lightweight construction in automotive engineering must meet complex requirements, however. It is essential to combine good formability with high strength in service, excellent corrosion resistance and weldability [1]. For automotive body panels, additional decorative requirements must be met, which require a perfect material surface. Figure 1 summarizes and ranks the property criteria for Al-Mg-Si sheets for various automotive body-in-white applications, which are irrespective of the variants of 6xxx alloys [2,3].

Heat-treatable alloys have the advantage of combining both good formability after solution-treatment and quenching and high strength after age hardening during the automotive paint bake process at ~185 °C. The paint bake process increases the strength of Al-Mg-Si alloys due to precipitation hardening and at the same time enables the curing of the paint [2].

Automotive engineers are demanding ever-higher strengths to generate greater potential for lightweight constructions. However, an increase in strength often involves a loss of ductility (formability). This is unsatisfactory for automotive designers, for whom excellent sheet product formability is a basic requirement, because automotive design must meet constantly increasing demands (e.g., sharp edges) [4]. Car manufacturers also require mechanical properties that remain stable for six months after solution heat treatment and quenching, to secure process stability during their forming operations. These conflicting needs must be balanced and require thorough knowledge of the interaction between material composition, production process and properties.

Silicon and magnesium are the main alloying elements of Al-Mg-Si alloys. In the last few years, several alloy modifications have been introduced to meet the requirements of car manufacturers. The 6xxx series alloys vary not only in their Si/Mg ratio; they also differ in their transition element additions (e.g., Cu, Mn, Fe and V). Different compositions in combination with specific processing modifications produce a wide range of mechanical properties in the final products.

The present study compares four prominent Al-Mg-Si alloys (AA6016, AA6005A, AA6063 and AA6013) with regard to the requirements of the automotive industry and illustrates the wide property range generated by changes in chemical composition and process parameters. It concludes by evaluating a broad range of test results and proposing uses for the investigated alloys.

## 2. Experimental Section

### 2.1. Alloy Production

The chemical composition of the investigated Al-Mg-Si alloys is shown in Table 1. The industrial alloys were cast as ingots and then scalped on their rolling surface. For hot rolling, the alloys were heated up to typical rolling temperatures. After this heat treatment, the hot ingots were transferred to the rolling line. Then, the hot band was coiled and allowed to cool before it was cold rolled to the final gauge of 1 mm. Solution treatment was performed in a continuous heat-treatment line, followed by subsequent water quenching. In addition to the simply quenched state (T4), a pre-ageing treatment (100 °C for 5 h) was carried out directly after solution treatment [5,6,7]. Within this investigation, the temper produced in this way is called T4*. Temper T4 and especially the pre-aged temper T4* are typical states delivered to the automotive industry, which generally exhibit good forming performance. The final strength of the manufactured parts is achieved after forming operations and via the automotive paint bake cycle, which is simulated within this work by a heat treatment of 20 min at 185 °C after 2% pre-straining. Temper T6 results from temper T4 followed by pre-straining for 2% and a heat treatment of 20 min at 185 °C. Temper T6* is similar to T6, but started from the pre-aged temper T4* condition.

**Table 1:** Chemical composition of the investigated alloys.

Alloy	Al (wt%)	Si (wt%)	Fe (wt%)	Cu (wt%)	Mn (wt%)	Mg (wt%)	Cr (wt%)	Zn (wt%)	Si/Mg
**AA6016**	Bal.	1.03	0.17	0.08	0.08	0.32	0.01	0.01	3.2
**AA6005A**	Bal.	0.81	0.19	0.04	0.17	0.50	0.01	0.01	1.6
**AA6063**	Bal.	0.64	0.19	0.02	0.02	0.55	0.01	0.02	1.4
**AA6013**	Bal.	0.75	0.27	0.75	0.50	0.98	0.01	0.02	0.8

### 2.2. Mechanical Properties

Mechanical properties in tempers T4, T4*, T6 and T6* are evaluated by tensile tests in testing direction LT (long transverse) according to EN ISO 6892-1 (L_0_ = 80 mm) [8]. The results of tensile tests are also of particular value for sheet metal forming operations. The tensile strain hardening exponent *n* was measured in accordance with ISO 10275 [9], and the vertical anisotropy *r* was determined in accordance with ISO 10113 [10] from tensile tests. To evaluate the plane anisotropy ∆*r*, the *r*-values were also measured in testing direction L, and 45° and ∆*r* was calculated according to Equation (1).

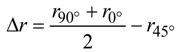
(1)


### 2.3. Forming Behavior

#### 2.3.1. Forming Limit Curve (FLC)

To describe the fundamental formability characteristics of the investigated sheet materials, a forming limit curve was carried out. The Nakazima test method was employed according to EN ISO 12004-2 (punch velocity: 1.5 mm/s; image rate: 15 Hz) [11]. Therefore, wasted blanks with a parallel shaft to the rolling direction were used. The blanks had different widths: 20 mm, 50 mm, 80 mm, 100 mm, 130 mm and a full-sized specimen.

#### 2.3.2. Bending Test

Bending performance was characterized by a plane strain bending test according to VDA 238-100 (within this study, only bending in the L-direction was investigated) [12]. The test was performed on sheet metal strips with a length of 250 mm (in the LT-direction) and a width of 70 mm (in the L-direction). The metal strips were pre-strained for 10% in the LT-direction and afterwards machined into the bending test samples of 60 × 60 mm^2^.

#### 2.3.3. Hole Expansion Test

Hole expansion tests were conducted on an Erichsen 142/40, 400 kN hydraulic sheet metal tester (Erichsen GmbH & Co KG, Hemer, Germany). Specimens for hole expansion testing were prepared and tested according to ISO 16630 [13]. The sheets were cut along the rolling direction into square specimens of 100 × 100 mm^2^. A conical punch with a top angle of 60° was used for hole-expansion purposes, and the initial hole was made by punching using a die diameter of 10 mm (*d*_0_). The motion of the punch into the hole was stopped manually when a crack at the edge of the expanding hole was observed. The limiting hole expansion ratio λ was then calculated according to Equation (2).

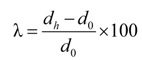
(2)


**Figure 2:**
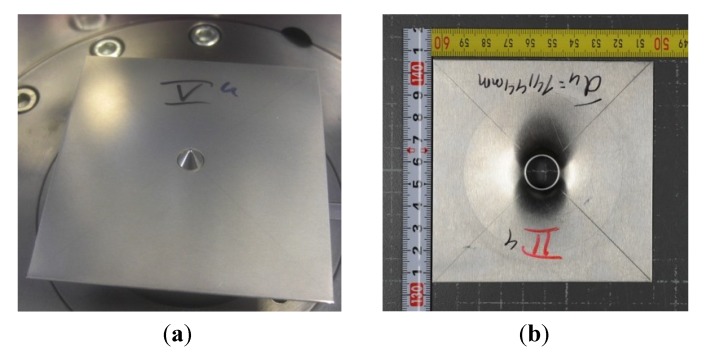
(**a**) Die tool used for the hole-expansion test; and (**b**) the final deformed part.

In Equation (2) *d_h_* (mm) is the average hole diameter after testing, and Figure 2 shows the die tool used for the hole-expansion test and a tested sample.

#### 2.3.4. Cross-Die Test

**Figure 3:**
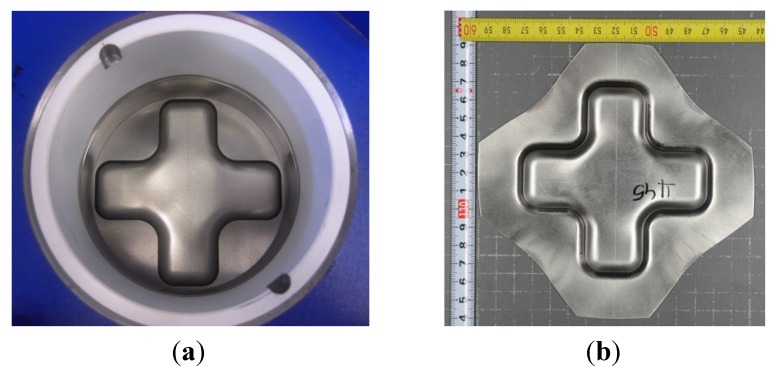
(**a**) Die tool used for the cross-die test; and (**b**) the final deformed part.

Cross-die tests are used for simulative testing by the automotive industry to evaluate the forming characteristics of sheet metals. Both the drawing height and the maximum strain, which can be sustained by the specimens before the onset of tearing, are delivered by the cross-die test. The experimental setup and the produced specimen are shown in Figure 3.

### 2.4. Surface Quality

**Figure 4:**
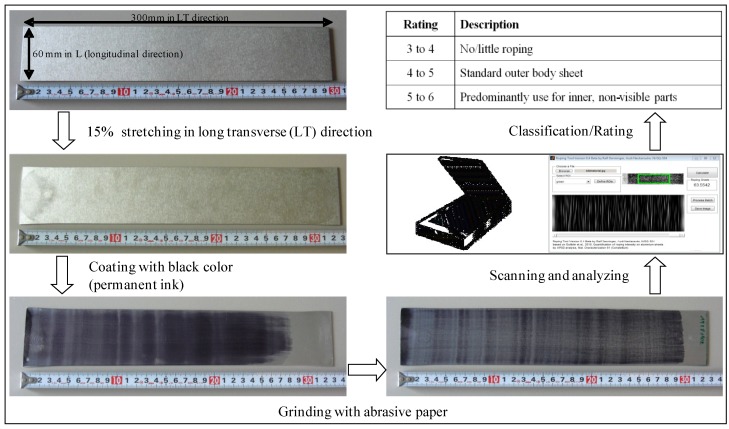
Roping test procedure rating.

Associated with automotive components are the deleterious surface defects referred to as roping, which appears on the surface of formed aluminum sheet components. Roping lines are present on the surface of the components as a series of closely spaced lines in the rolling direction (L). The roping lines appear in the rolling direction only when sufficient transverse strain is applied. For testing, a sample perpendicular to the rolling direction (LT) was stretched with a permanent elongation of 15% in a tensile testing machine (Zwick GmbH & Co KG, Ulm, Germany). For systematic analysis, the sample was then painted black on both sides with a fine-pored sponge. Next, the sample was ground with P800 grade abrasive paper. The grinding process was carried out manually in the LT-direction at a low grinding pressure. After this procedure, any existing roping lines are clearly visible. To classify the samples in this respect, they were computer-scanned with a picture resolution of 300 dpi. Afterwards, the scanned pictures were imported into the image processing software Audi Roping Tool v.054 (Audi AG, Neckarsulm, Germany), which automatically calculates the roping grade with an inaccuracy of ~10%. The procedure and the classification criteria are illustrated in Figure 4.

### 2.5. Corrosion Behavior

Intergranular corrosion (IGC) testing was performed according to ISO 11846 method B [14]. In preparation, samples were etched twice: for 90 s in hot caustic soda solution and for 60 s in a hydrofluoric acid containing etching solution, as described in the standard. The classification criteria for this test method are shown in Table 2. Samples were immersed for 24 h at 30 °C in a solution containing 30 g/L NaCl and 10 mL concentrated hydrochloric acid. The type and depth of the corrosion attack were evaluated by an optical microscope on 30 mm-long metallographic cross-sections longitudinal to the rolling direction.

**Table 2:** Classification of intergranular corrosion. IGC, intergranular corrosion; PC, pitting corrosion.

Grade	Type of Corrosion	Acronym
1	Pitting corrosion	PC
2	Pitting and intergranular corrosion (dominant PC)	PC/IGC
3	Intergranular and pitting corrosion (dominant IGC)	IGC/PC
4	Intergranular corrosion: local	IGC4
5	Intergranular corrosion: quasi area-wide	IGC5

### 2.6. Microstructure Observation

Samples for scanning electron microscopy (SEM) and energy dispersive X-ray spectroscopy (EDX) were prepared by mechanical grinding and polishing. SEM characterization of the microstructure was done by a Zeiss EVO 40 microscope (Carl Zeiss GmbH, Jena, Germany) equipped with a tungsten cathode and employing an acceleration voltage of 15 kV and a working distance of 6 to 8 mm. The chemical composition of the constituents was determined by EDX on polished surfaces of the samples. For light optical micrographs, an Olympus AHMT-3 microscope (Olympus GmbH, Hamburg, Germany) was used.

## 3. Results

### 3.1. Mechanical Properties

**Figure 5:**
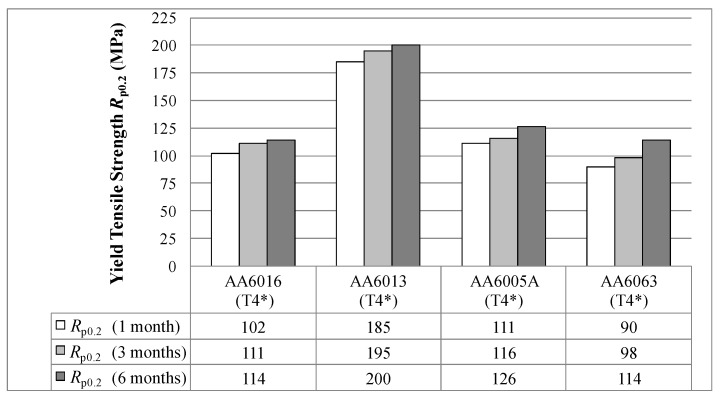
Yield tensile strength *R*_p0.2_ in temper T4* measured one, three and six months after sheet production (LT-direction).

Figure 5 shows the yield strength (*R*_p0.2_) in temper T4* after different storage periods following sheet production. In general, the yield strength increases with storage at RT. AA6013 exhibits the highest *R*_p0.2_ of 185 MPa in temper T4* after one month after sheet production. The yield strength of AA6013 increases over six months from 185 to 200 MPa. In contrast, AA6063 shows the lowest *R*_p0.2_ of 90 MPa after brief room temperature storage, but exhibits the highest overall strength increase over the storage time. The yield strength of AA6016 stays nearly constant after three months and shows the smallest overall increase when stored at RT.

**Figure 6:**
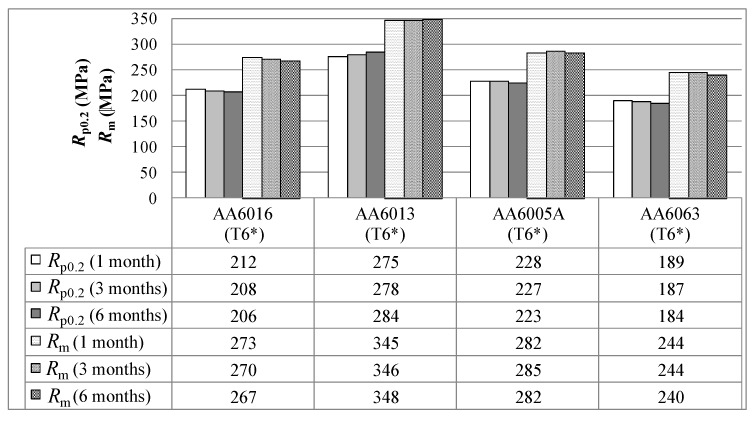
Yield tensile strength *R*_p0.2_ and ultimate tensile strength *R*_m_ in temper T6* measured one, three and six months after sheet production (LT-direction).

Figure 6 summarizes the mechanical properties of the investigated alloys after pre-aging and the simulated paint bake process (termed T6*). The achieved yield strength decreases with increasing storage time. This was observed for all alloys except AA6013, which showed a slight increase in strength over time. It was found that AA6005A generates a superior yield strength of 223 MPa after a six months storage time in comparison to AA6016 and AA6063. Overall, AA6013 exhibits the highest yield strength of 275 MPa after one month of storage. Ultimate tensile strength results are also shown in Figure 6, as these values are of interest for the materials in service. However, the general trends among the alloys appear to be similar.

**Figure 7:**
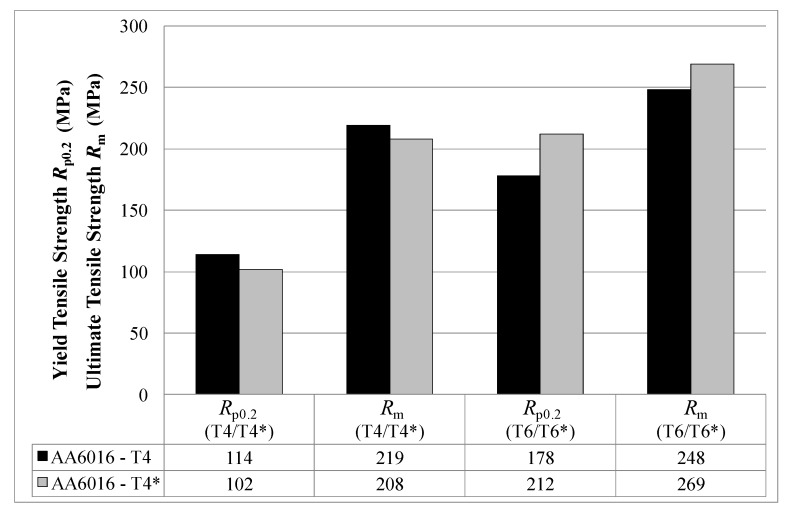
Yield tensile strength *R*_p0.2_ and ultimate tensile strength *R*_m_ in tempers T4, T4*, T6 and T6* measured one month after sheet production (LT-direction).

In order to show the influence of pre-aging on mechanical properties, Figure 7 illustrates the yield strength and ultimate tensile strength of alloy AA6016 for temper T4 (without pre-aging) and temper T4* (with pre-aging) after one month of storage and in the resulting tempers T6 and temper T6*, respectively. T4 values are generally higher than temper T4*, and a simulated paint bake process produces a more pronounced increase in strength after pre-aging.

**Figure 8:**
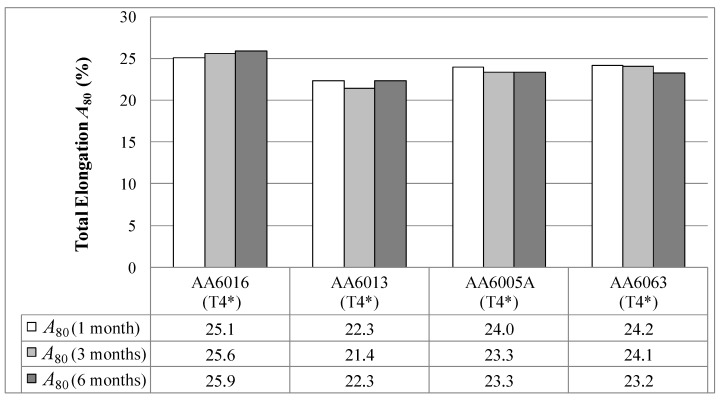
Total elongation *A*_80_ in temper T4* measured one, three and six months after sheet production (LT-direction).

Figure 8 illustrates the total elongation *A*_80_ of the investigated alloys. AA6016 reaches the highest elongation value of nearly 26% after six months of storage time. AA6013 exhibits the lowest elongation. The results show only small changes over storage time for all of the alloys.

**Figure 9:**
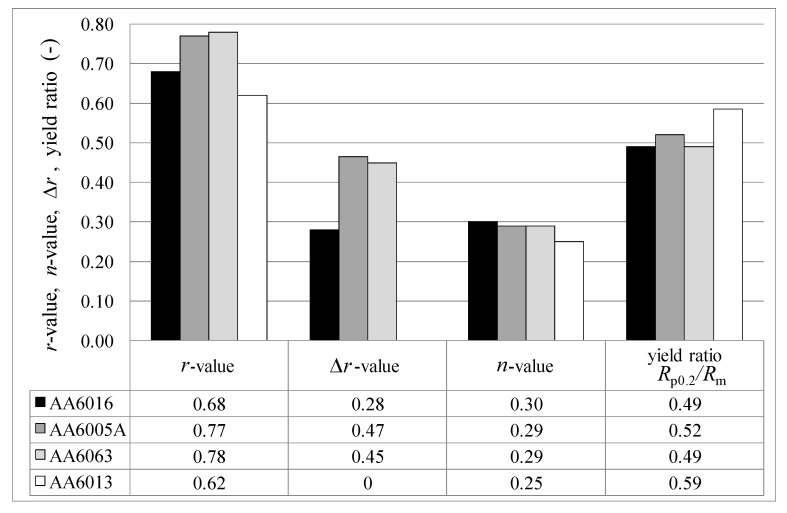
Vertical anisotropy *r* (LT), strain hardening exponent *n* (LT), plane anisotropy *∆r* (according Equation (1)) and *R*_p0.2_/*R*_m_ ratio (LT) in temper T4* measured one month after sheet production.

Figure 9 shows the vertical anisotropy *r*, the plane anisotropy ∆*r*, the strain hardening exponent *n* and the yield ratio *R*_p0.2_/*R*_m_ for a storage period of one month after sheet production for the investigated alloys as deduced from tensile tests. All shown values are nearly constant after one month of storage. AA6005A and AA6063 reach a high *r*-value in 90°, but the plane anisotropy of these alloys is high, which means that the forming behavior is not uniform in all rolling directions (0°, 45° and 90°). AA6016 provides a relatively low ∆*r*-value of 0.28 and shows the highest *n*-value of 0.30 of all of the alloys. AA6013 exhibits the most uniform *r*-value in all rolling directions, with a plane anisotropy of zero. The yield ratios *R*_p0.2_/*R*_m_ of the investigated alloys are nearly identical, with the exception of AA6013, which shows a comparably high yield ratio of 0.59.

### 3.2. Forming Behavior

#### 3.2.1. Forming Limit Curve (FLC)

**Figure 10:**
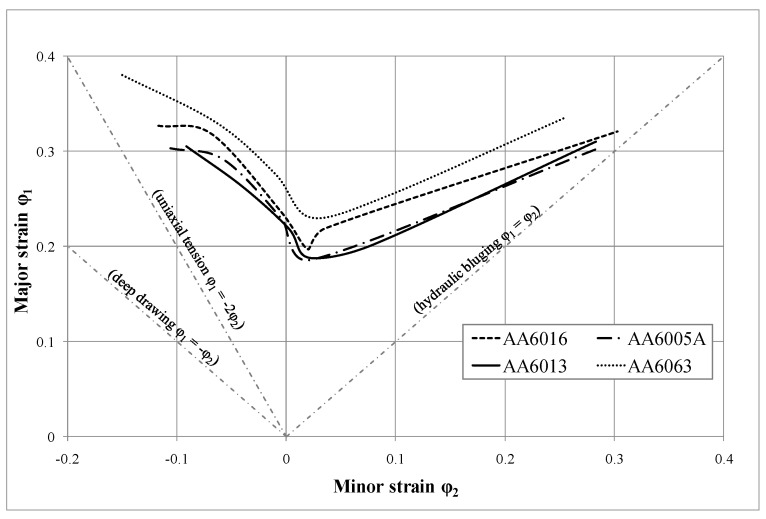
Forming limit curves of AA6016, AA6005A, AA6063 and AA6013 in temper T4* for a sheet thickness of 1.0 mm measured two months after sheet production.

Figure 10 shows that AA6063 allows the greatest deformation before the first failures occur, and that the forming limit curves (FLCs) of AA6013 and AA6005 appear to be rather similar at lower strain values. The FLC of AA6016 lies between those of the other alloys.

**Figure 11:**
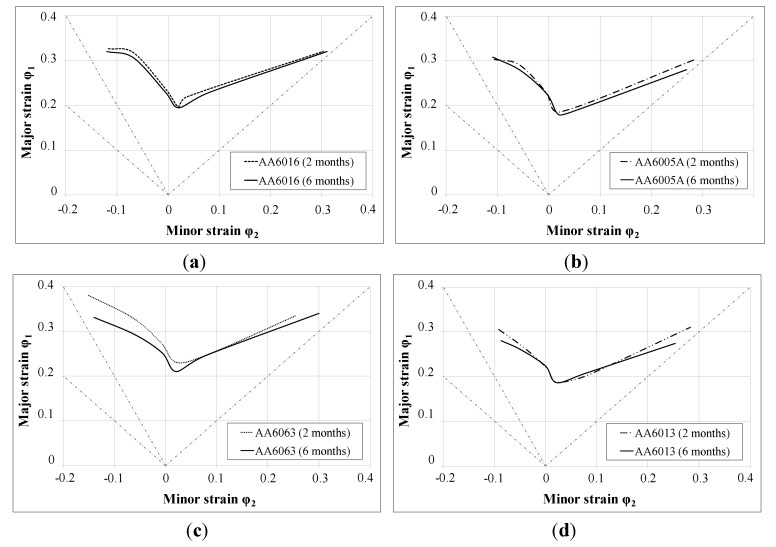
Forming limit curve of (**a**) AA6016; (**b**) AA6005A; (**c**) AA6063; (**d**) AA6013 in temper T4* for a sheet thickness of 1.0 mm measured two and six months after sheet production.

Figure 11a–d illustrates the forming limit curves of the investigated alloys as a function of room temperature storage time. The curves were measured two and six months after sheet production. The FLC of the tested alloys show only a weak decrease due to storage at RT. Alloy AA6063 showed the strongest deviation between two and six months of storage time.

#### 3.2.2. Bending Performance

Table 3 depicts the bending results for the investigated material after different storage periods. AA6016 and AA6063 exhibit the best bending performance, with a bending angle of nearly 150° even after six months of storage at RT. In contrast, AA6013 shows the weakest bending performance with a bending angle of around 80°. It was found that the bending performance of AA6016 stays nearly constant at the value reached after three months’ natural ageing, whereas the bending behavior of AA6063 shows a more pronounced dependence on RT storage.

**Table 3:** The bending angle (in °) measured one, three and six months after sheet production in temper T4*.

Age	Bending Angle (°) of different alloys			
	AA6016	AA6005A	AA6063	AA6013
1 month	156	134	159	84
3 months	152	128	155	83
6 months	151	126	147	77

#### 3.2.3. Hole-Expansion Test

Table 4 lists the limiting hole-expansion ratio λ of the investigated alloys for various storage periods. Alloys AA6016, AA6005A and AA6063 show nearly the same results with a ratio >50%. Alloy AA6013 reaches the lowest hole-expansion ratio, around 41%. The results show only slight changes over storage time for all alloys, but by a trend, the hole expansion ratio decreased with RT storage.

**Table 4:** Limiting hole-expansion ratio (in %) measured one, three and six months after sheet production in temper T4*.

Age	Limiting hole expansion ratio λ (%) of different alloys			
	AA6016	AA6005A	AA6063	AA6013
1 month	53.0	56.7	52.3	41.6
3 months	52.3	55.3	52.7	42.0
6 months	52.0	55.3	51.3	41.3

#### 3.2.4. Cross-Die Test

**Figure 12:**
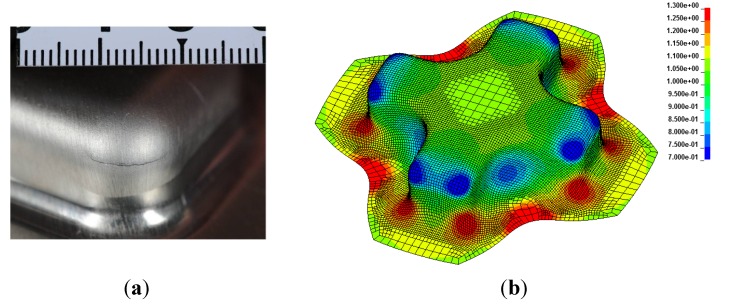
The appearance of a crack due to thinning on the corners of the cross-die sample: (**a**) experiment and (**b**) simulation of the material thinning in (mm).

Before the experimental test was carried out, a simulation was performed to predict where and at what forming height the first cracks can be expected. The simulation result was verified to agree well with the experiment. The first cracks occur due to thinning in the corners of the formed cross (Figure 12b illustrates this in blue). Due to thinning, the material thickness decreased in this case from 1.1 mm to 0.7 mm. In this area, the material is mainly stretched under biaxial tension.

The forming height of the investigated alloys is given in Table 5 as a function of storage time. It was found that alloys AA6005 and AA6016 achieve the best results in this test at a forming height of around 18 mm, whereas AA6013 only reaches a height of around 15 mm. The results showed a slight decrease in height with increasing storage time.

**Table 5:** Cross-die forming height (in mm) measured one, three and six months after sheet production in temper T4*.

Age	Cross-die forming height different alloys (mm)			
	AA6016	AA6005A	AA6063	AA6013
1 month	18.2	18.4	17.9	15.3
3 months	18.1	18.2	17.2	15.0
6 months	18.2	17.9	16.6	14.7

#### 3.2.5. Surface Quality

**Figure 13:**
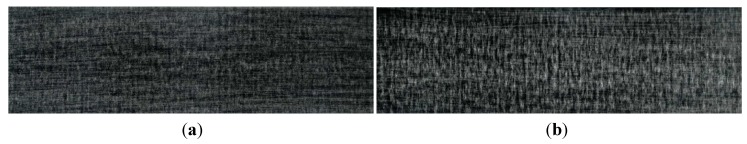
Roping behavior: (**a**) no/little roping; (**b**) clearly visible roping.

Figure 13 illustrates roping test samples after tests on a material with low (Figure 13a) and high roping sensitivity (Figure 13b).

Table 6 summarizes the results of the roping test. The roping behavior was analyzed after different storage periods, but storage does not influence the classification. It was found that AA6016 generates the best roping result with a rating of 3.8 (no/little roping), followed by AA6005A and AA6063. Only AA6013 reaches an insufficient value (5.4).

**Table 6:** Classification of the roping test samples.

Classification	Classification rating of different alloys			
	AA6016	AA6005A	AA6063	AA6013
Roping behavior	3.8	4.2	4.5	5.4

#### 3.2.6. Corrosion Behavior

**Figure 14:**
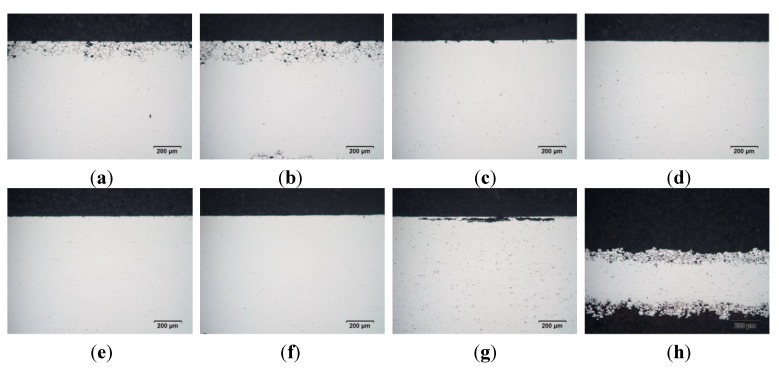
Corrosion behavior in temper T4* and temper T6*. (**a**) AA6016–1.10 mm–T4*; (**b**) AA6016–1.10 mm–T6*; (**c**) AA6005A–1.00 mm–T4*; (**d**) AA6005A–1.00 mm–T6*; (**e**) AA6063–1.15 mm–T4*; (**f**) AA6063–1.15 mm–T6*; (**g**) AA6013–1.02 mm–T4*; (**h**) AA6013–1.02 mm–T6*.

Table 7 and Figure 14 present the corrosion test results for the investigated material. Testing was performed on samples that were naturally aged for one month. AA6016 exhibits IGC (see Table 4 for corrosion modes) with a maximum corrosion depth of 150 µm in temper T4* (Figure 14a). Alloys AA6005A and AA6063 are nearly unaffected in temper T4*. Only a few attacks can be observed in the surface region, as shown in Figure 14c,e. AA6013 shows PC (pitting corrosion) in temper T4* with a maximum depth of 50 µm (Figure 14g). Different behavior was seen in condition T6*. After the simulated paint bake treatment, AA6013 exhibits IGC with a corrosion depth of 350 µm (Figure 14h). AA6016 was also found to be susceptible to IGC in temper T6*, but with a more moderate maximum corrosion depth of 180 µm (Figure 14b). However, AA6005A and AA6063 show no attack in the T6* temper (Figure 14d,f).

**Table 7:** Classification of corrosion behavior in temper T4* and temper T6*.

Sample	Alloy	Temper	Type of corrosion	Depth (µm)
a	AA6016	T4*	IGC, Grade 5	150
c	AA6005A	T4*	Low IGC/PC, Grade 3	30
e	AA6063	T4*	Low IGC, Grade 4	15
g	AA6013	T4*	PC, Grade 1	50
b	AA6016	T6*	IGC, Grade 5	180
d	AA6005A	T6*	No attack	0
f	AA6063	T6*	No attack	0
h	AA6013	T6*	IGC, Grade 5	350

## 4. Discussion

### 4.1. Mechanical Properties

In general, solution heat-treated and quenched tempers are non-stable tempers. Strength increases with storage time due to natural ageing, and this is attributed to the clustering of solute atoms (Mg, Si and also Cu, if contained in the alloys) [15]. Pre-aging treatments can decrease hardening during natural ageing (clustering) due to a decrease of the concentration of quenched-in vacancies and the formation of larger clusters [16,17]. This explanation is assumed to be the reason why the initial strength level of T4 is higher than for T4* (see Figure 7). However, further atom probe analysis would be necessary to explain this more precisely.

Pre-aging has been shown to reduce the effect of natural ageing, but it cannot be fully prevented during RT storage (see Figure 5). The automotive industry must ensure quality restrictions for at least six months. Within this period, mechanical properties must remain within the specification limits and variations should be as low as is physically possible. For this purpose, the most “stable” of our alloys is AA6016 (in terms of absolute values), which does not change its yield strength significantly upon long-term storage (Figure 5).

The 6xxx series aluminum alloys are characterized by their main alloying elements, Si and Mg, and the additions of Cu, Mn and Fe. As Table 2 shows, the alloys studied exhibit different Si/Mg ratios. AA6016 shows the highest and, in consequence, also a high amount of Si in excess of the equilibrium precipitate phase Mg_2_Si [18], which forms in Al-Mg-Si alloys. It is known that a high Si/Mg ratio produces an increased strengthening coefficient, which improves the formability of the alloy [19]. This effect was also observed in the present study. Accordingly, AA6016 reaches the highest *n*-value, whereas AA6013, which exhibits a higher amount of Mg than Si, exhibits a lower *n*-value (Figure 9). Due to the observed lower elongation of AA6005A and AA6063 in comparison to AA6016, a lower Si/Mg ratio may also generate lower elongation values (Figure 8).

Alloy AA6013 was found to exhibit better strength levels than the other alloys in tempers T4* and T6*. This is simply because of its chemical composition. Adding copper to Al-Mg-Si alloys refines the precipitate structure, induces the strengthening phase Q’ (Cu-containing phase) and, therefore, increases the strength level [20]. The relatively high Mn-content in AA6013 was also shown to increase its initial strength, presumably due to solid solution hardening [21]. In contrast, AA6063 provides the lowest strength, both in tempers T4* and in T6*. This alloy contains nearly no Cu and Mn, and the Mg and Si content is also comparably low.

The higher T6* strength of alloy AA6005A compared to AA6063 can be dedicated to its higher content of hardening elements. Although the amount of Mg + Si is comparable in AA6005 and AA6016, AA6005 offers a higher T6* strength. This might be related to its well-balanced Si/Mg ratio. It is assumed that because of the close compositional relationship between alloy AA6005 and the major hardening precipitate (probably β'' = Mg_5_Si_6_ [22]), a higher number density is formed, which, in turn, generates higher strength in T6* temper [23]. Note that the paint bake response of the investigated alloys is better if pre-ageing was carried out directly after solution annealing (compare T6 and T6* in Figure 7). It is assumed that the Mg,Si clusters formed during the pre-aging with subsequent natural aging exceed a critical size, which makes them more stable than those in the naturally aged T4 state [24]. The stable clusters can act as nuclei for the β'' precipitation during subsequent artificial aging and, therefore, enhance the artificial aging kinetics [16]. We have discussed T4* and T6* tempers in more detail than tempers T4 and T6, because T4* and T6* are more important conditions for future applications.

Anisotropy plays an important role during forming processes and can lead to earing during deep drawing. Some characteristics of anisotropy can already be deduced from tensile tests, as shown in Section 3.1. Materials with a high vertical anisotropy *r*-value possess high resistance to plastic flow in the direction of the sheet thickness. The strain-hardening exponent *n* can be considered as an indicator of the maximum attainable deformation during cold forming. The higher the n-value and, consequently, the higher the uniform strain, the lower the tendency of the material to neck locally. AA6013 shows the most uniform *r*-value in all rolling directions, with a plane anisotropy of zero (Figure 9). Normally the *r*-value in the 45° direction is much lower than the values for 0° and 90° in Al-Mg-Si alloys, but AA6013 exhibits a very high *r*-value at 45° to the rolling direction of 0.78. Because *r*-values correlate with the rolling texture, it can be assumed that the high Cu-content and the low Si/Mg ratio in AA6013 influence the rolling texture positively. Further, texture investigations would be necessary to address this result more precisely.

### 4.2. Formability

Using forming limit curves (illustrated for the investigated alloys in the T4* temper in Figure 10 and Figure 11), it is possible to determine process limitations in sheet metal forming in the case of a linear strain path (e.g., necking and tearing). AA6063 provides the most promising FLC, which is presumably related to its lower strength level and lower amount of main alloying in comparison to the other alloys (Figure 5). Although AA6013 shows the highest strength during tensile testing (Figure 5), the FLC is almost comparable to AA6005A. A detailed explanation of this result is outside the scope of this paper, but a correlation with the very low plane anisotropy *∆r* of AA6013 (due to the high *r*-value at 45°) is assumed. In general, FLCs are not strongly influenced by RT storage after sheet production, which is a rather satisfying result in view of the requirements of the automotive industry (Figure 11). However, alloy AA6063 showed the strongest, though moderate, influence of storage, which can be related to the yield strength increase upon storage, which was also highest for AA6063 (Figure 5).

In addition to the Nakazima tests (FLC), hole-expansion tests, bending tests and deep-drawing tests in a cross-die were conducted to characterize the forming behavior of the materials investigated in this study. Test procedure limitations make it necessary to carry out more than one forming test to analyze the formability of a sheet metal and to predict its behavior during industrial forming processes.

Hemming is a typical assembly method used in the automotive industry to join the outer sheet to the inner part of hang-on body panels. The requirements on an alloy that is subjected to hemming are tough, because the material has to withstand strong bending over a radius equal to half of the sheet thickness. Therefore, high aluminum sheet bendability is a desirable property in the production of an automotive body panel. It has been shown that a high yield strength in temper T4* generates low bendability [25,26]. Failure during bending of Al-Mg-Si alloys can occur through intergranular fracture due to the presence of grain boundary particles [27]. Strain localization and intense shearing in relation to micro-void formation around large phase particles can also cause fracture during bending [28]. AA6016 and AA6063 showed the best bending performance (they exhibit the lowest *R*_p0.2_ in T4*; see Figure 5) compared to AA6005A and AA6013 (Table 3). The poor bending performance of AA6013 may be related, on the one hand, to its Cu-content: copper tends to form grain boundary precipitates. On the other hand, large intermetallic particles can also influence the bendability negatively [19]. Industrially produced Al-Mg-Si alloys always contain iron. This governs the formation of intermetallic Al-Fe-Si-(Mn) particles, which influence the formability negatively. In Al-Mg-Si alloys, β-AlFeSi and α-AlFeSi particles are mainly present. These particles form during solidification and homogenization of as-cast ingots and do not dissolve when the alloy is further heat-treated. The α-particles have a more globular morphology than the plate-like β-particles, which are known to promote the formation of voids during deformation [29,30,31,32]. AA6013 and AA6005A contain more Fe and Mn, which also matches the observed trend in the bending angle (Table 3).

**Figure 15:**
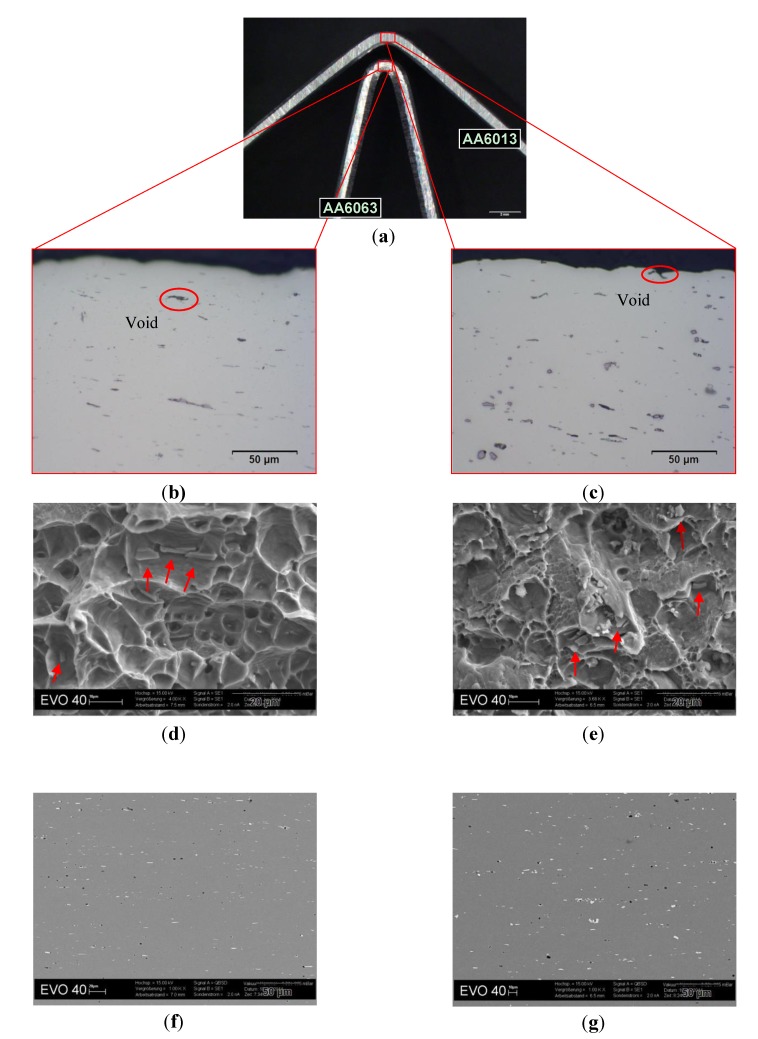
Microstructure observations on bending test samples of AA6063 and AA6013 in T4*. (**a**) Bending samples of AA6063 and AA6013 in temper T4*; (**b**) optical micrograph in the L-direction of AA6063 in T4* near the fracture surface, 500×; (**c**) optical micrograph in the L-direction of AA6013 in T4* near the fracture surface, 500×; (**d**) SEM micrograph of the fracture surface of AA6063 in T4*, red arrows = Al-Fe-Si, 4,000×; (**e**) SEM micrograph of the fracture surface of AA6013 in T4*, red arrows = Al-Fe-Si-Mn-Cu 4000×; (**f**) SEM micrograph of the undeformed microstructure of AA6063 in T4*, 1,000×, white = Al-Fe-Si particles dark = Al-Si-Mg particles; (**g**) SEM micrograph of the undeformed microstructure of AA6013 in T4*, 1,000×, white = Al-Fe-Si-Mn-Cu particles black = Al-Mg-Si-Cu particles.

To strengthen the above-mentioned correlations, Figure 15 shows a comparison between AA6063 (good bendability) and AA6013 (poor bendability) in terms of microstructure. For this observation, micrographs were taken from bending samples (Figure 15a). Figure 15b,c show the microstructure near the fracture surface of the bending samples. It was found that AA6063 exhibits a more homogenous microstructure in comparison to 6013. Alloy AA6013 shows large particles of high density, mainly Al-Fe-Si-Mn-Cu constituents. Cracks appeared due to void formation in regions of maximum strain. To illustrate fracture surfaces (Figure 15d,e), the samples were bent until complete fracture occurred. AA6063 showed a ductile, homogenous forced fracture. In contrast, AA6013 was again more inhomogeneous (shear fracture areas were observed). To investigate the microstructure of both alloys in more detail, also the undeformed material was analyzed (Figure 15f,g). AA6063 showed less constituents and more plate-like Al-Fe-Si particles (6063 has nearly no Mn, which would be required to promote the formation of globular α-AlFeSi). In comparison AA6013 exhibited more globular particles (due to the high Mn-content), but showed an inhomogeneous and denser distribution of large constituents.

The design requirements of automotive parts often demand the presence of holes on the surface. Because hole-expansion tests measure the elongation of the material near the holes, calculating the hole-expansion ratio is of great interest in generating information about the material’s susceptibility to edge cracking or edge stretching. The stress field in the formed edge where the cracks appear is similar to the stress field in the sheared edge during flanging operations. The edge condition before flanging, sheet-deformation during hole preparation, punch shape and microstructure (volume fraction and morphology of different phases) can affect the ability of the hole flange to stretch [33]. The alloy AA6013 shows the lowest hole expansion ratio, around 41% (Table 4), which was predicted due to the high strength level of this alloy in temper T4* (Figure 5). AA6013 also contains high amounts of alloying elements, which form different intermetallic phases (e.g., Al-Fe-Si-Mn, Mg-Si, Al-Cu-Mg-Si) with a high volume fraction through the thermo-mechanical processing. These phases can cause grain boundary precipitations (mainly Cu-rich precipitates). Fracture during deformation can then be initiated in the grain boundary through void initiation [34].

The forming test in a cross-die has been used extensively in the automotive industry to assess the formability of sheet material. The geometry of the cross-die displays typical stress states, which predominate during the manufacture of real automotive parts. Qualitatively the results of this test, summarized in Table 5, compare well with the results of the hole-expansion test (Table 4).

Overall, the forming results were found to depend on the chemical composition of the alloy and the specific testing procedure used. For example AA6013, which is widely used in the aerospace industry, showed poor forming performance during bending and hole-expansion, probably because of its very high strength level in temper T4*. However, despite its high strength, AA6013 was almost competitive in the other forming tests.

### 4.3. Surface Quality

An important consideration for sheets, which are used in outer car body panels, is their surface appearance after the final forming process. The 6xxx series alloys are known to show roping (also called ridging) after stretch forming. This phenomenon occurs on the surface of aluminum sheets during forming operations in the transverse direction (LT). The ridges formed are up to 30 µm in depth and several centimeters in length, and they are arranged in a longitudinal direction (rolling direction, L). Roping is still visible after the surface of the formed sheets is painted. Such optical defects cannot be tolerated in outer skin applications. Physically, roping is caused by bands of similar crystallographic grains oriented in a preferred direction. Various studies state the predominant factors in roping to be the initial texture and the spatial distribution of grain orientations [35,36,37,38,39]. Roping behavior can be enhanced with a special heat treatment during the rolling process. Such treatment randomizes the distribution of grain orientations and, therefore, reduces the roping phenomena [40,41,42]. In this study, the investigated alloys were analyzed in terms of roping (Section 3.2.5). The applied roping test is currently used by German car manufacturers to classify outer body sheets. It was found that alloys AA6016, AA6005A and AA6063 fulfill the requirements of the outer skin material (Table 6). Only alloy AA6013 shows insufficient roping behavior for body panels.

### 4.4. Corrosion Behavior

Nearly every car manufacturer has its own extensive long-term corrosion test procedure for analyzing the corrosion behavior of aluminum sheet alloys. In this study, intergranular corrosion (IGC) tests were performed according to ISO 11846 method B [14]. This testing method makes possible the relatively quick classification of different Al-Mg-Si alloys. Here, several car manufacturers require a maximum IGC-depth of 300 µm for body panels. Intergranular corrosion of 6xxx series alloys has been attributed to the depletion of Si and/or Cu along grain boundaries [43]. Susceptibility to IGC is, in general, strongly dependent on the thermal history during fabrication. For Al-Mg-Si alloys containing Cu, it has been shown that the formation of a nano-scaled Cu-enriched layer along the grain boundaries due to ageing contributes to IGC. It has been assumed that the layer serves as a precursor of the Q phase [44]. With increasing ageing temperature and time, the layer transforms into Q precipitates, and the IGC susceptibility decreases. Precipitated Si on grain boundaries is also claimed to act as a local cathodic site in alloys containing excess Si [43]. In this study (Table 7 and Figure 14), it was found that AA6013 is highly susceptible to IGC after the simulated paint bake process. This was no surprise, because of its relatively high Cu content and the fact that paint bake treatment is relatively brief. Therefore, the above-mentioned Cu-layer at the grain boundaries is assumed to remain present in T6* temper. The good corrosion resistance of AA6063 and AA6005A in comparison to AA6016 can be related to their low Si/Mg ratio, which goes hand in hand with a low amount of Si in excess. However, the results of corrosion tests on AA6016 would be sufficient for its use in outer car body panels, because the automotive manufacturers require a maximum IGC-depth of 300 µm, and AA6016 exhibits a maximum IGC-depth of 180 µm.

## 5. Conclusions

An investigation into typical automotive Al-Mg-Si sheet alloys with regard to the property criteria crucial in the automotive industry has inspired the following proposals regarding their use:

- Alloys AA6016 and AA6005A showed a well-weighted property profile for use in outer body panels. They exhibited excellent bendability, moderate strength in temper T4*, high strength in service (T6*), good corrosion resistance and the best surface quality with regard to roping;
- Alloy AA6063 is very suitable for decorative inner parts, which require excellent formability during extensive deep drawing. Slight, well-defined changes in its chemical composition and/or processing route might increase the in-service strength level of this alloy, which would also make it suitable for outer body panels;
- The high-strength alloy AA6013 would be an excellent candidate for structural parts, which only require moderate formability. Unfortunately, the alloy showed a susceptibility to IGC due to its high Cu-content; this might be minimized by special heat treatment (e.g., 205 °C/30 min);
- The influence of room temperature storage after sheet production on most properties is of marginal concern. AA6016, in particular, performs well in terms of long-term storage stability.

We have provided these criteria for the use of AA6016, AA6005A, AA6063 and AA6013 in the automotive industry by considering holistic aspects, such as mechanical properties, forming and corrosion behavior and achievable surface quality in service.
